# Dermatological diseases from a genetic perspective

**DOI:** 10.1515/medgen-2023-2009

**Published:** 2023-04-05

**Authors:** Regina C. Betz, Ulrike Hüffmeier

**Affiliations:** University of Bonn Institute of Human Genetics Bonn Deutschland; Institut für Humangenetik Erlangen Deutschland

Since the publication in 2009 of the previous *Medizinische Genetik* issue on genetic skin and hair diseases, exciting discoveries have been made in the field of dermatogenetics. As with other disease phenotypes, the increasing use of new high-throughput sequencing and array-based technologies has led to the discovery of new genes and new gene loci, as well as the consideration of novel therapeutic approaches. In this issue, five research groups highlight the latest developments in their respective areas of expertise, which relate to diverse monogenic and genetically complex disorders of the skin and hair.

Skin disorders rank among the most common and visible of all human diseases, and OMIM contains more than 2,250 items for disease entities affecting the skin and its accessory structures. From eczema and psoriasis to albinism and the alopecias, genetic dermatological disorders range from those that are mild and manageable to those that are severe and even life-threatening. Overall, the field of dermatogenetics focuses on two broad categories of dermatological conditions, which can be distinguished on the basis of their underlying genetic architecture: 1) rare monogenic diseases, such as ichthyoses, rare hypotrichoses, blistering diseases, keratinisation disorders, and ectodermal dysplasias; and 2) common genetically complex diseases, such as atopic dermatitis, male pattern baldness (MPHL; androgenetic alopecia), alopecia areata (AA), and common psoriatic manifestations.

In monogenic skin and hair diseases, advances in next generation sequencing (NGS) technologies have led to the genetic elucidation of multiple disorders. These NGS technologies encompass both whole exome- and (less frequently) whole genome sequencing. Notably, these technologies have elucidated the genetic background of numerous individual and familial cases that could not have been clarified via linkage analysis. In this issue, the review of syndromic forms of ichthyosis by Fischer et al. discusses a number of new syndromes that have been identified via these technologies in recent years. It is important to note, however, that many subtypes with a presumed monogenic background still await genetic elucidation. The relative lack of progress for these subtypes may be attributable to factors such as limited sample sizes, as well as difficulties associated with identifying pathogenic variants that are located in non-coding regions, or those that have reduced penetrance and higher allele frequencies in the population. Nonetheless, advances in medical technologies and comprehensive genetic testing continue to facilitate the diagnosis of monogenic disorders. Accurate diagnosis is a crucial first step for patients and their families, as it enables clinicians to provide a more accurate prognostic assessment with regards to expected signs and symptoms, including those that may only manifest in later life.

Equally impressive progress has been made for genetically complex phenotypes. In the case of MPHL, for example, the article by Henne et al. explains that while only two gene loci were known in 2009, nearly 400 risk loci have now been identified. On the one hand, this is due to the assessment of larger collectives and the possibility of collaboration with large national cohorts (e. g. UK Biobank). On the other, this progress can be attributed to technological advances, and a substantial reduction in the respective costs (e. g. for microarrays). Similarly, genome wide association studies (GWAS) have led to an enormous increase in our understanding of AA, as summarized in the article by Basmanav and Betz. For eczema (atopic dermatitis), almost 60 susceptibility loci have been detected to date, as outlined in the article by Marenholz et al. A proportion of these loci overlap with those identified for other, related allergic manifestations, and the identification of protein-altering variants at ~20 % of loci is high compared to the percentage identified for other genetically complex diseases. For example, at the more than 80 susceptibility loci identified for the common psoriatic subtypes psoriasis vulgaris and psoriatic arthritis, protein-altering variants have only been identified for single loci/pathways, as reviewed in the article by Klima et al. Of note, both articles highlight the fact that single genes from GWAS have been shown to harbour rare disease-causing variants in single families/cases and vice versa. In the next few years, elucidation of the role of rare variants in common dermatological diseases will represent a key research focus in dermatogenetics.

These advances in our understanding of complex forms of dermatological disease open avenues for clinical translation. In recent years, polygenic risk score (PRS) for genetic risk modelling has been developed, and several studies have shown that this may represent a promising approach within clinical practice for disease phenotypes such as cardiovascular disorders. Potentially, PRS could also be implemented in routine clinical practice within dermatogenetics and other clinical specialties in coming years. For MPHL, Henne et al. explain that the tested prediction models have yielded what may be considered moderate accuracy, but that these models require improvement, if they are to deliver more reliable and accurate predictions, particularly on the individual level. Research assessing the prediction accuracies of PRS in other genetically complex skin and hair phenotypes such as AA, eczema, and psoriasis is ongoing, and the results are expected in the near future. Initial studies on the efficacy of treatments for psoriasis also suggest that genetic risk factors can help to assess response rate, e. g. for biological therapeutic agents targeting interleukin pathways.

Thanks to modern treatments and interventions, individuals with genetically determined dermatological disorders can now live fulfilling and active lives, e. g. through the prescription of JAK inhibitors in atopic dermatitis, or diverse biological therapeutics in psoriasis. Notably, the detection in 2011 of a deficiency of the receptor antagonist in the IL-36 pathway in a rare psoriatic subtype led to the later development of an IL-36 receptor blocker. A recent pioneer study showed that this medication was a highly efficacious treatment for this psoriatic subtype.

For many dermatological disorders, there is still a long way to go in terms of both understanding causal factors and developing effective treatments, and despite encouraging developments in dermatogenetic research, many gaps remain in our knowledge of the mechanisms that underlie skin disorders. Therefore, further functional research into both rare and common skin and hair disorders must be vigorously pursued. Transcriptomic datasets have proven valuable resources in terms of deciphering disease pathobiology, identifying molecular biomarkers that can be informative of disease activity, and monitoring a patient’s response to medication at the molecular level, as in the case of AA (e. g. Alopecia Areata Disease Activity Index). In the future, the integration of different layers of high-throughput omics data (e. g. genomics, epigenomics, and proteomics) from affected tissues- or single-cells will facilitate elucidation of the regulatory and complex landscapes of these disease entities.

We hope that the following articles on recent genetic insights into these diverse disorders will refresh your knowledge, and contribute to an improved understanding of the genetic basis of dermatological diseases.

